# Labels Affect Food Choices, but in What Ways?

**DOI:** 10.3390/nu14153204

**Published:** 2022-08-05

**Authors:** Swen J. Kühne, Ester Reijnen, Gracinda Granja, Rachel S. Hansen

**Affiliations:** School of Applied Psychology, ZHAW Zurich University of Applied Sciences, Pfingstweidstrasse 96, P.O. Box 707, CH-8037 Zürich, Switzerland

**Keywords:** food label, Nutri-Score, sugar, healthy food, calorie, averaging bias, licensing effect

## Abstract

To reduce obesity and thus promote healthy food choices, front-of-pack (FOP) labels have been introduced. Though FOP labels help identify healthy foods, their impact on actual food choices is rather small. A newly developed so-called swipe task was used to investigate whether the type of label used (summary vs. nutrient-specific) had differential effects on different operationalizations of the “healthier choice” measure (e.g., calories and sugar). After learning about the product offerings of a small online store, observers (*N* = 354) could, by means of a swipe gesture, purchase the products they needed for a weekend with six people. Observers were randomly assigned to one of five conditions, two summary label conditions (Nutri-Score and HFL), two nutrient (sugar)-specific label conditions (manga and comic), or a control condition without a label. Unexpectedly, more products (+7.3 products)—albeit mostly healthy ones—and thus more calories (+1732 kcal) were purchased in the label conditions than in the control condition. Furthermore, the tested labels had different effects with respect to the different operationalizations (e.g., manga reduced sugar purchase). We argue that the additional green-labeled healthy products purchased (in label conditions) “compensate” for the purchase of red-labeled unhealthy products (see averaging bias and licensing effect).

## 1. Introduction

When grocery shopping, *unhealthy food products* tempt you everywhere—such as the Snickers at the checkout. While you probably know that the Snickers is unhealthy, people’s health knowledge of food products is often incorrect. For example, Lucky Charms are perceived as healthy cereals [1]. Therefore, these “unhealthy” products often end up in the shopping basket without a guilty conscience [2], thus contributing to the worldwide increase in overweight and obesity [3]. In this context, the nutritional information (nutrition facts) printed on the back of food packaging seems to do little to solve the problem, as consumers perceive them as *too complex* [4]. Moreover, the brief glimpse, if any, that consumers give to the nutrition facts (e.g., 0.7 s in a grocery store study, see Bartels [5]) is not enough to make sense of the information. For this reason, so-called front-of-pack labels or FOP labels, such as the “Guideline Daily Amounts” (GDA) or the “Traffic Light Label” (TLL), have been developed to provide nutrition or health information in a *simple* and quick way.

According to Ikonen et al. [6], current FOP labels (note that FOP labels are voluntary in many countries—an exception is Chile where one type is mandatory [7]—but they are usually recommended by the authorities as in France, Spain, and Switzerland [8]) fall into the following three categories (see Elmadfa & Meyer [9], for an alternative classification system): reductive labels, nutrient-specific (interpretive) labels, and summary indicator labels. The first category of labels simply *reduces* the information overload of the nutrition facts (e.g., GDA). Accordingly, this category of so-called *reductive labels* does not include an interpretive component; this is in contrast to the labels of the other two categories. The second category of (*nutrient-specific*) labels, such as the TLL or the Chilean warning label, interpret the health value of specific food nutrients (e.g., salt). Finally, the third category of (*summary indicator*) labels interpret the overall health value (more precisely the nutritional value) of the food product in summary form (e.g., Nutri-Score). However, the classification of labels into the latter two categories is not always clear-cut. The Chilean warning label, for example, is sometimes also classified as a summary indicator label [10].

Categorization helps us navigate the label jungle by helping us to understand which categories of labels are best for *identifying* healthy foods and, more importantly, whether this improved ability to recognize healthy foods then also leads to healthier food choices or *purchase intentions.* Accordingly, the meta-analysis of 114 studies conducted by Ikonen et al. [6] showed that mainly interpretive summary indicator labels (such as the Nutri-Score label) have a strong positive effect on the identification of healthy foods. However, the effect of labels—regardless of type—on healthier food choices is rather small. While other recent meta-analyses and reviews [9,11,12] confirmed this small effect, Cecchini and Warin [13] reported much larger effects. This is probably because the latter authors worked with a smaller sample of studies (9 rather than the 114 in Ikonen et al. [6]). The finding that the *better identification* of healthy products does not automatically lead to them being *chosen or purchased* is reminiscent of the so-called knowledge–behavior gap [14], which means that although people are aware of the problematic consequences of their behavior, they often do not change it. In the energy sector [15], for example, people are aware of the rise in temperature due to CO_2_ emissions caused by fossil fuel consumption but do not buy green electricity. As it turns out, this gap cannot be closed in nutrition either, even if the relevant information (e.g., in form of labels) is made available at the time of the decision.

What might explain these *small effects* found in relation to “healthier food choices”? One part can be explained by the different outcome measures or operationalizations that were used to measure different *aspects* of the underlying construct. What do we mean by that? In the energy sector, for example, a “greener behavior” can be achieved by reducing the *total energy consumption* (reducing kWh) or by *using renewable energy sources* (e.g., solar and reducing CO_2_ emissions). However, since both operationalizations measure a slightly different aspect, the latter does not necessarily lead to a reduction in total energy consumption. Accordingly, one finds different results depending on the operationalization used. Now, in nutrition, with respect to measuring healthier food choices, various operationalizations have been used, such as reducing a specific nutrient or calorie content. All of them measure slightly different aspects. To the other part, and this complicates matters further, certain labels or label categories presumably show an effect only with respect to certain outcome measures. This means that some FOP labels could lead to a reduction in the consumption of certain nutrients, such as sugar or salt, while others may make people eat healthier on average. Understanding the interplay or dependence between FOP labels and operationalizations or outcomes of healthier food choices is important from a scientific perspective, as this has not yet been systematically studied. For example, although Neal et al. [16] used different operationalizations, they did not test the Nutri-Score. However, its understanding would also be important for policy makers. It could help them to choose the right label for the problematic consumption behavior. For example, a sugar label on sugar-sweetened beverages to reduce their consumption.

**Figure 1:**
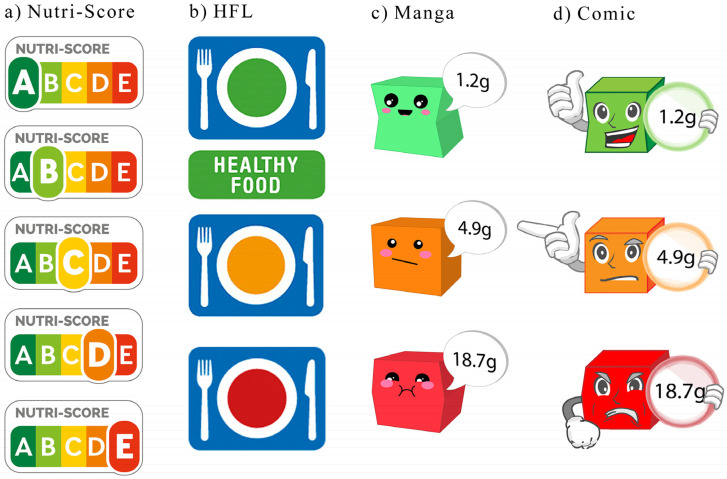
The four different labels used: (**a**) the Nutri-Score, (**b**) the HFL, (**c**) Manga, (**d**) Comic. The last three labels were newly developed. Whereby the HFL is an adoption of the Nutri-Score with just three levels. The Manga and Comic focus on the Sugar content.

What operationalizations (or outcome measures) have been studied so far, especially regarding interpretive labels (i.e., nutrient-specific and summary indicator — we focus on interpretative labels in our study, as reductive labels showed no benefit in the meta-analysis conducted by Ikonen et al. [6])? One of them is the reduction of a *specific nutrient,* such as salt, in the diet (see for example, McLean et al. [17]). In that regard, the Chilean warning label that indicates products with a high sodium content, such as chips, with the words “high in sodium” successfully reduced the purchase of such products (see Taillie et al. [18], for results on other nutrients). However, choosing or buying fewer products that are high in a specific nutrient (e.g., salt) does not automatically lead to an overall healthier diet or even weight loss. For weight loss, a reduction in *calorie intake* is crucial [19,20]. In this regard, the meta-analysis by Croker et al. [9] showed that, again, labels indicating that a food product is “high” in something, in this case calories, reduced the purchase of these products. However, calories do not consider, for example, the negative effects of salt [21] and/or the positive effects of fiber [22]. Therefore, the so-called *FSA score* and, accordingly, its reduction might be better operationalizations [23,24]. The FSA score combines the positive effects or attributes of a food product, such as proteins, dietary fiber, fruits, vegetables, and seeds, and the negative effects or attributes, such as calories, saturates, sugar, and sodium, into a single score [25]. The score ranges from −15 to +40, with a low score (below zero) representing a high nutritional value and a high score (more than 19) representing a low nutritional value. Diets with high FSA scores are associated with health issues (e.g., higher mortality [26]). Since the FSA score encompasses several aspects of healthy eating, it is currently considered one of the best operationalizations. Various summary labels (the third category in the taxonomy of Ikonen et al. [6], such as the Health Star Rating System, the NuVal, and the Nutri-Score label, have been developed, and the Nutri-Score uses the FSA score as a basis. In the Nutri-Score label, on which we focus, the FSA score enables the classification of food products into five health categories ranging from A (green = highest nutritional value) to E (red = lowest nutritional value; see Figure 1a). Because the Nutri-Score label has the FSA score as its basis, the assumption is that it will be more successful than other labels, such as the TLL. This has been tested by Dubois et al. [27], among others. They applied one of four possible FOP labels, including the Nutri-Score label and TLL, to products from four different categories (fresh ready meals, bakery products, bread, and canned ready meals). Each label was thereby tested in 10 supermarkets in France. The so-called control condition consisted of 20 supermarkets in which the corresponding products were not labeled. Unexpectedly, however, none of the tested labels led (compared to the control condition) to a significant reduction of the *average FSA score* in the shopping cart. This was despite 14.4% more healthy foods being purchased under the Nutri-Score label.

As can be seen, different operationalizations were used, and some of them were also tested with respect to several labels, although not yet systematically. Therefore, the obtained results are inconclusive. In this study, we aim to systematically investigate the interplay between labels and outcome measures. At the same time, we want to address some shortcomings of the labels tested so far and improve them by redesigning them. What are these shortcomings? Let us first start with the already described summary indicator label, the Nutri-Score label with its five categories. Although the use of categories or “categorizing” makes human life easier by reducing the workload, the number of categories used in tasks can be significant. For example, Dallet [28] found that word list recall is best when the number of categories is four or less. We wondered, therefore, whether a label with only three categories and thus more similar to a classic traffic light, which normally contains three different colored lights, would be more advantageous, not to mention that the “in-between” category B, for example, of the Nutri-Score label, is quite narrowly defined, and accordingly only a few products fall into this category [29]. Therefore, a new label, the so-called Healthy Food Label (HFL; Figure 1b), was designed (for more details see the Method Section). Furthermore, for nutritional information to be processed both *quickly* and easily, color or its use is of central importance (for information about the impact of colors in a visual search task [30,31]). Hence, Antúnez et al. [32] have shown that colored labels, such as the TLL, as opposed to monochromatic labels, can indeed draw attention more quickly to the relevant information (e.g., high fat content; 3225 ms vs. 964 ms). In addition, the meaning of the colors green, orange, and red used in the TLL color code system seems to be well-understood by consumers [33]. Finally, the TLL colors affect food choices more than other categorical systems, such as, the use of other colors (e.g., blue, white, and purple) or smileys [34]. Although the Nutri-Score label and other labels tested here use colors, this is rarely the case for *nutrient*-specific (interpretive) labels. Hence, we wondered if such labels, such as the Chilean warning label combined with the traffic light system, would be more effective in guiding people to reduce consumption of the targeted nutrient (e.g., salt). Therefore, new labels (called manga and comic) using the TLL (the color-coding is based on the recommendations of the WHO of 50 g of sugar per day, which is 10% of the energy intake [35]. 0–5% of the recommended amount of sugar = green color, 6–25% = orange color, and more than 25% = red color) color coding were designed for the target nutrient sugar (for more details see the Method Section). We chose the nutrient sugar because a reduction in sugar content is one of the aspirational goals of the Swiss government in collaboration with companies such as Nestlé and Wander [36]. Moreover, a previous study [37] showed that the sugar content of a product is a key component in food choices. Finally, sugar labels have been shown to be quite effective in guiding consumers to products with lower sugar contents [38].

In summary, in this study we investigated the relationship between labels and different operationalizations. Thereby, the newly developed labels (HFL, manga, and comic) were tested against the Nutri-Score label and a control condition without a label. Based on the labels and the chosen outcome measures, the question can be answered as to which labels (if any) are more beneficial (summary indicator or nutrient-specific) and in relation to which outcome measure (e.g., sugar reduction only). This was tested in a newly developed online shopping procedure.

## 2. Method

### 2.1. Participants

A total of 354 participants aged 19 to 46 years old (*M*_age_ = 24.77; *SD_age_* = 4.43; 62.4% female) from ZHAW Zurich University of Applied Sciences and the greater area of Zurich (3%) took part in this smartphone-based online study. Regarding income, 60% of participants earned less than 2000 CHF per month. As an incentive, participants could enter a raffle for one of two iPads (which a total of 86.4% did) or, if they were a student of the ZHAW School of Applied Psychology, they could receive course credit instead (which 4.8% overall did). All participants gave informed consent.

### 2.2. Stimulus Material

**Figure 2:**
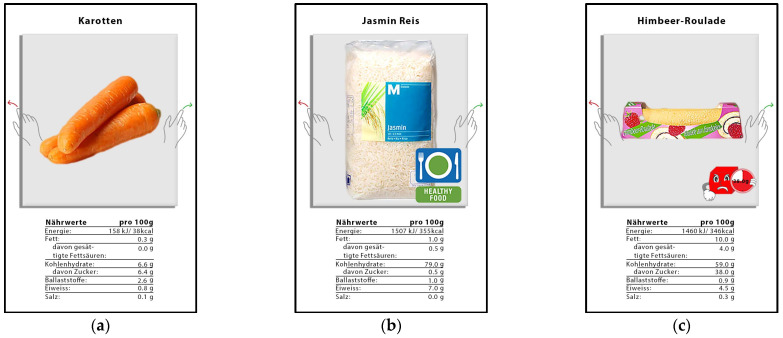
Examples of some selected conditions of the shopping task: (**a**) without label, (**b**) with the HFL, and (**c**) with the comic label.

As stimulus material, we used pictures (taken with permission from the online store of one of the largest Swiss retailers) of 100 different foods (for simplicity, we use the term foods, but we also included some beverages) from nine different categories: beverages, bread, meat, dairy and eggs, veggie (vegetables and fruits), pasta (pasta, rice, and pasta sauce), snacks, sweets, and frozen food (see Appendix B Table A1 for detailed information). The selected foods should represent the diversity of a small grocery store. The pictures were supplemented with the names as well as the nutritional facts (the nutrition facts were shown to exclude the possibility that any FOP label effect is only due to a lack of information in the control group or condition) of the foods (see Figure 2a). Depending on which condition participants were randomly assigned to, the food picture contained either a label (i.e., Nutri-Score label, HFL, manga, or comic; see Figure 1b,c for some examples) or none (control condition). The Nutri-Score label is described in detail in the Introduction. The other *summary indicator* label is the newly developed Healthy Food Label (HFL,designed with the help of two designers—U. Binder and Th. Gfeller—from the ZHdK Zurich University of Applied Arts; Figure 1b). Like the Nutri-Score, the HFL is based on the FSA score, but categories A and B of the Nutri-Score are combined into one category with a green label, and categories D and E are combined into one category with a red label. The middle category C of the Nutri-Score is also the middle category in the HFL and is therefore given an orange label. Hence, the HFL has three categories. Note that the green label also contains the words “healthy food”. The other two labels, also newly developed (designed by members of the team), are two nutrient-specific labels (called manga and comic; see Figure 1c,d) that indicate the sugar content of a product using the TLL color system. The labels symbolize cubes with faces or a facial expressions. The face of the green labels looks cheerful, and its shape is rather thin. The faces of the orange labels are grumpy, and their shape is a bit puffier. The faces of the red labels are sad (manga) or angry (comic) and thick in shape. Faces or facial expressions were added to make them more visible (although basic research implies some benefits of faces in capturing attention [39,40,41], studies have not found that, for example, smileys are better than colored labels [34]), especially for children, on which they will be tested in the future.

### 2.3. Procedure

As an introduction to the shopping task, participants had to imagine that it was Friday evening and that—since unexpected guests had been announced for the weekend—they had to buy groceries for six people at a small nearby store that was still open. Since people usually know what products are available in a particular store, we familiarized (*familiarizing task*) our participants with the store’s food products before they performed the actual *shopping task*. We did this by showing them its 100 products, at nine product pictures per page, without any nutritional information. To make sure they paid attention, they had to mark (by tapping) all the products they had bought at some point in their lives. Thereafter, participants were randomly assigned to the different conditions of the shopping task, with an additional brief introduction to the respective label in the label conditions. In the shopping task, they were again presented with the 100 products, but this time, one after the other in a random order. They could then buy or not buy the presented food with a swipe gesture to the left or right (similar to the dating app Tinder but with food as the stimulus, see Figure 2). A similar “swipe” task was also used in some of the studies by Park and Simonson [42] to simulate purchase decisions (e.g., for paintings). To again guarantee that participants paid close attention to the foods, 10 control displays, each showing two food images side by side, were interspersed at random locations. The participant’s task in this regard was to indicate (by a swipe gesture) which of the food products (left or right) they had seen immediately before. After the shopping task, the participants were presented with some questions. For example, participants in the label conditions were asked if they had paid attention to the respective labels when making purchases and, if so, whether that had helped them buy healthier or less sugary foods. The participants of the control group (the no label condition) were asked which of the four labels would best help them to buy healthier or less sugary foods. At the end of the study, all participants’ demographic data (e.g., age and sex) were assessed.

Regarding the shopping task, the following outcome measures were assessed: the total number of food products purchased, the total number of healthy products (A and B categories of the Nutri-Score and the green category of all other labels) purchased, the total and mean FSA scores, the total and mean number of calories, and the total and mean amounts of sugar of the products purchased. We therefore analyzed all the products that ended up in the shopping basket at the end of the shopping task. The FSA score, calories, and sugar were thereby calculated based on 100 g per product, as no size information (in g or l) about the food products was provided to the participants. An alternative would be to scale values at the effective product size (e.g., 75 g for chips). However, as a package size was not indicated to the participants and the FSA score is designed as a measurement of the nutritional value per 100 g, scaling the product size would not be more accurate. The outcome measures were collected both across all categories and specifically per category.

## 3. Results

### 3.1. Participants Excluded

From the 417 participants that completed the study, 11 participants (2.6%) who needed more than 60 or less than 6 min to complete the study, were excluded from the analysis. Furthermore, 33 participants (7.9%) who answered only 60% or less of the control questions correctly were excluded. Finally, four participants who participated twice in the study and one participant who selected only one product were excluded.

### 3.2. Descriptive Statistics

Table 1 provides an overview (i.e., means and standard deviations) of the different outcome measures per condition. It appears that participants in the control (no label) condition had lower scores in all the “total” scores but similar scores in the “mean” scores compared to the label conditions.

### 3.3. Inferential Statistics

Regarding each outcome measure, we first conducted an ANOVA across the aggregated data (i.e., across all categories) to test for an overall effect of label. In case of a significant result, a planned contrast of “label vs. no label” and Tukey-adjusted post hoc tests between the label conditions were calculated. Subsequently, the factor “categories” was added to the ANOVAs to test for each outcome measure whether labels (i.e., indicated by the “label vs. no label” contrast) had different effects across food categories. A note on the data handling: If a participant did not select a food product within a category, we coded this with the value 0. While this does not affect the results of the analysis of the total number of (healthy) products purchased, it could be the case regarding the total and mean values for FSA score, calories, and sugar. To verify if this was the case, we replaced the missing values with “NA” and reanalyzed the data in two different ways, namely, calculating either ANOVAs with listwise deletion (imputation procedures did not seem feasible, as participants actively did not choose any product in the category) or ANOVAs without repeated measures (thereby ignoring within-subject variance). Overall, the two methods showed similar results in terms of all “total” outcome measures, even at the category level (i.e., when we included the factor “categories” in the ANOVAs). Unfortunately, a less stable pattern was found regarding the “mean” outcome measures, mainly at the category level (i.e., the label vs. no label contrast became insignificant when the category factor was added). Therefore, we decided not to report those values. This is indicated in Table 2 with “N/A” in the corresponding cells.

**Figure 3:**
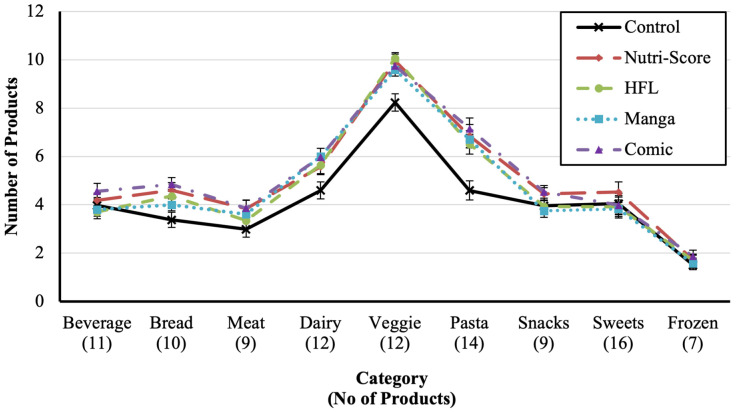
Number of products bought per category.

We see, for example (to illustrate the results in Table 2 or Figure 3), that the total number of food products purchased was significantly higher in the control condition (no label) than in the conditions with labels. However, a closer look at the post hoc tests shows that this effect is mainly due to the labels *Nutri-Score* and *comic*. These appear to lead to significant increases in the food products purchased in the bread, dairy, veggie, and pasta categories (all *t* > 3.06, *p* < 0.01) but not in the other categories (all *t* < 1.83, *p* > 0.06). Regarding the total number of healthy food products, on the other hand, all labels were advantageous. Furthermore, label effects were found in all categories (all *t* > 2.36, *p* < 0.05), except in the category sweets, as there were no healthy sweets. In terms of the other measures, we found mixed results. For example, in terms of total calories, only the comic label had an effect. Thereby, we found an effect in the same categories as the total number of products purchased. While the Nutri-Score only affected the number of (healthy) products, the other labels further affected either the total or the mean, the FSA score, calories, or sugar. For example, the comic label led to a reduction in total calories, while the HFL lead to a reduction in the mean FSA score. For detailed results, see Appendix A.

### 3.4. Questions

Regarding the label conditions, we asked participants if they used the labels in the shopping task, and 43% of the participants in the Nutri-Score, 34% in the comic, 30% in the HFL, and 27% in the manga label conditions reported that they used the labels. Moreover, in all label conditions, around 90% of the participants recognized what the labels stood for. In the *control condition* most participants stated that the Nutri-Score label would be the most helpful label for choosing healthy products (62%), ahead of the HFL (33%) and the two sugar labels (comic 14% and manga 7%). In terms of sugar reduction, they indicated that the comic label (49%) would be the most helpful, ahead of the manga label (23%), Nutri-Score label (26%), and HFL (9%). Interestingly, participants in the different *label conditions* (i.e., Nutri-Score, HFL, manga, and comic) stated, independent of the condition they were in, that their respective label helped them to buy not only healthier but also less sugary foods. Hence, they did not differentiate between the labels like the participants in the control group.

## 4. Discussion

**Figure 4:**
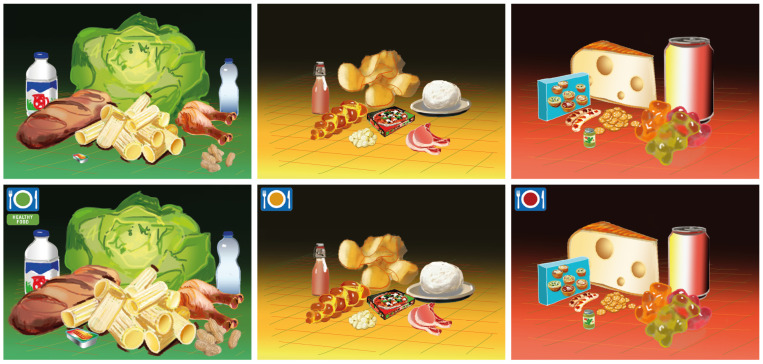
Illustration of the foods purchased in a comparison of the HFL vs. control. This is a “still life” illustration of the number of products purchased in each category, separated by their healthiness (e.g., green table = healthy). Sizes illustrate how many products were purchased in the category. For example, the chicken leg represents all healthy meats purchased by its size, and the bratwurst represents unhealthy meats purchased. Illustrations were created by Ulrich Binder and Simon Truffer.

Our results indicate that *overall*, “labels” made participants buy on average about *7.3 more products*, resulting in a higher total FSA score (+110 points) and higher total calories (+1732 kcal). The additional products purchased, however, were mainly healthy products (+5.9 products) from the categories of vegetables, fruits, bread, dairy, eggs, pasta, pasta sauce, and rice (see Figure 4). That those additional products were mostly healthy is also reflected by the 0.72 points lower mean FSA score in the label conditions.

Our results broken down by the *individual* labels show that the Nutri-Score label led people to buy more products, which were, in general, healthy products but also included rather unhealthy products. This contrasts with the study of Dubois et al. [27], where the Nutri-Score label only increased the purchase of healthy products. Furthermore, we did not find a significant difference between the Nutri-Score and the control group in the mean FSA score. This is in contrast to the small difference of 0.14 FSA points reported by Dubois et al. [27] and the larger difference of 2.65 FSA points reported by Corsetto et al. [43]. The large difference in the study by Corsetto et al. [43] could have been due to the fact that participants had to choose twice from the same product range, once without and once with a label. This could have led to an overestimation of the label effect. Dubois et al. [27] thereby had a larger sample size compared to our study and could detect such small label effects.

The HFL did not lead people to buy healthy products *instead* of unhealthy ones (indicated by the number of products chosen, whereby the number of healthy foods chosen increased by six due to the HFL, and the number of products chosen overall also increased by 6, although the later post hoc test was not significant). Hence, both the Nutri-Score and the HFL only make people add mostly healthy products, such as an apple, to the existing (unhealthy) dietary pattern. Now consider Julia et al. [44], who calculated that a one point increase in the mean FSA score of a diet is associated with a 16% increased risk of obesity in men. This means that labels only lead to an overall healthier diet if the additional healthy foods purchased are integrated into the regular daily consumption (i.e., the purchase lasts longer) and not simply added to the foods usually consumed. For example, in the study by van den Akker et al. [45], labels such as the Nutri-Score led to healthier product choices and more accurate serving sizes. This indicates that labels do not lead to a rebound effect (choosing healthier options but eating more food), as sometimes speculated in the literature. Otherwise, the tested labels might even accelerate the obesity crisis. This might sound like a harsh conclusion, which of course must first be replicated in the field.

What about the sugar labels? Although the comic label (like the Nutri-Score) resulted in an increase in the total products purchased and total calories, like the manga label, it resulted in an increase in the total healthy products purchased and, more importantly, a decrease in the mean sugar purchased (−1.0 g). This means that the products in the basket contained less sugar on average. Interestingly, as with all the “total” outcomes (see the other labels), the total sugar content was not reduced, again likely due to the increased number of products purchased. Surprisingly, the sugar labels did not lead to a decrease in the number of purchased sweets or beverages—categories for which we expected such labeling would be particularly effective [12]. The sugar reduction thus appears to be of a more general nature. In summary, we found some effects of labels as well as differential effects of labels on different outcome measures (e.g., sugar labels lead to a reduction in sugar consumption). However, none of the labels were able to reduce the purchase of unhealthy foods such as sweets or snacks. To our knowledge, a reduction in such unhealthy foods has not been observed in other studies with a shopping task (sugar sweetened beverages seem to be an exception, see Temple [12]).

This leaves us with the question: why do people buy more healthy products but not fewer unhealthy ones? Or more precisely, what *cognitive mechanism* could explain this behavior? In this regard, let us look at the study by Wansink et al. [46]. They showed that consumers whose shopping carts were divided into healthy and unhealthy food sections bought more vegetables and fruits than consumers whose carts were not divided. It is believed that when consumers become aware that the section for unhealthy foods in their cart is already quite full, they will add healthy foods to lower the “unhealthy” food average in their cart. The labels in our study might have led to that same effect (see Appendix A for more details). Hence, consumers display the so-called “averaging bias”, which assumes that they “balance out their evaluations using an averaging rather than an additive rule” (p. 745, Chernev & Gal [47]). That is, although the average FSA score (or calories) has been lowered—by adding healthier foods—the overall content of the shopping cart has become higher in FSA (or calories) and thereby rather unhealthier. This “averaging bias” was also demonstrated in a study by Forwood et al. [48] in which observers perceived the combination of an unhealthy food (burger) and a healthy food (celery sticks) to be relatively healthier (or lower in calories) than the unhealthy food (burger) alone. Similar effects have been reported in several food-related studies [47,49] as well as in relation to carbon emission estimates [50,51]. According to Chernev and Gal [47], this bias is also the cognitive antecedent for the motivational licensing effect, which has also been found in the food domain [52,53]. It is commonly assumed that an expected negative affect, for example, the expected feeling of regret when buying an unhealthy candy bar, regulates the purchase, and we might not buy the bar [54]. In terms of “motivational licensing”, this negative feeling could now be compensated by also buying a healthy apple or adding other healthy foods to the shopping basket. To our knowledge, a licensing effect in the context of food labeling has not been observed so far, but that emotions can play a role in food shopping combined with labels was shown by Thunström [55]. She thereby showed that calorie labels evoked good feelings in people with rather good self-control, but negative feelings in the minority of people with rather low self-control. Therefore, people with good self-control adjusted their behavior (had a reduced WTP for high-calorie foods) more than those with lower self-control. However, further experiments are needed to test whether motivational licensing is responsible for the results observed in this study.

Before we conclude, we would like to address some possible limitations of this study: First, in supermarkets, consumers have the opportunity to first obtain an overview of the product range in a certain category before they look more closely at a few selected products to make a purchase (see [56,57] for research in that field). We tried to imitate this situation as well as possible by first showing our participants all the products available in the store (familiarization task) and only then letting them decide (shopping task) which products they wanted to buy. However, this new process requires further evaluation. Second, it could be argued that the task of shopping for groceries for six people for a weekend does not depict a representative shopping behavior and therefore was not understood by the participants. However, the number of products purchased (*M* = 43.1, *SD* = 16.6) suggests that the participants understood the task. It might be the case that people buy groceries differently for themselves compared with buying groceries for others, but because all conditions had the same task, we can legitimately draw inferences about the labels’ effects on choice among the conditions.

## 5. Conclusions

Labels, especially the HFL, seem to be able to boost healthy food product sales. However, whether this is beneficial to consumers cannot be concluded from the results of our study, as more products and calories were purchased overall. If consumers would eat all the food they bought in the same timeframe as without labels, this would not lead to a reduction in their calorie intake. Therefore, the effect of food labels on real food intake should be addressed by future studies using multiple adequate outcome measures. So far, it seems that labels affect food choices, but the effects are smaller and not as clear as estimated in the past. This could be partly because—as already mentioned—the underlying mechanisms are not yet sufficiently understood.

## Figures and Tables

**Table 1:** Total and mean scores of the chosen outcome measures (or operationalizations) per condition.

Condition		Control (*N* = 69)	Nutri-Score (*N* = 65)	HFL (*N* = 70)	Manga (*N* = 79)	Comic (*N* = 71)
Outcome measure		*M (SD)*	*M (SD)*	*M (SD)*	*M (SD)*	*M (SD)*
Total						
	Number of products	37.3 (17.0)	45.73 (17.0)	43.23 (15.2)	42.93 (15.4)	46.53 (17.3)
	Number of healthy products	19.73 (8.4)	26.1 (8.4)	25.6 (6.9)	24.5 (6.9)	26.1 (8.1)
	FSA score	714.5 (363.6)	849.4 (359.0)	785.1 (341.3)	793.0 (336.8)	868.5 (371.2)
	Calories	8791.2 (4797.9)	10,891.0 (4766.4)	10,031.8 (4517.2)	10,123.1 (4332.8)	11,044.5 (4827.9)
	Sugar	273.1 (182.4)	312.7 (168.6)	281.0 (154.6)	262.7 (158.6)	283.7 (162.0)
Mean						
	FSA score	18.8 (2.1)	18.3 (1.7)	17.7 (2.2)	18.1 (1.9)	18.3 (1.9)
	Calories	228.8 (28.4)	233.0 (29.5)	224.4 (37.8)	231.4 (28.4)	231.5 (28.4)
	Sugar	6.8 (2.2)	6.6 (2.1)	6.2 (2.1)	5.8 (2.2)	5.8 (2.0)

To ensure that there are no negative score values regarding the FSA score, the score was adjusted upward by 15 points. Red indicates negative effects of a label (e.g., more products purchased compared to the control), and grey indicates positive label effects (e.g., lower mean FSA score than the control group).

**Table 2:** Results of the ANOVAs for the different outcome measures (or operationalizations).

Outcome Measure		ANOVA Across Categories *F*(4, 349)	“No Label vs. Label” Contrast across Categories *t*(349)	Sign. Tukey-adj. Post Hoc Tests between “No Label” and “Any Other Label” *t*(349)	Sign. “No Label vs. Label” Contrast within Food Categories *t*(349)
Total					
	Number of products	3.39 ** (*η^2^* = 0.04)	3.32 ***	Nutri-Score, Comic	bread, dairy, veggie, pasta
	Number of healthy products	8.62 *** (*η^2^* = 0.09)	5.72 ***	Nutri-Score, HFL, Manga, Comic	all (but sweets)
	FSA score	2.02 . (*η^2^* = 0.02)	2.30 *		bread, dairy, veggie, pasta
	Calories	2.56 * (*η^2^* = 0.03)	2.78 *	Comic	bread, dairy, veggie, pasta
	Sugar	0.88	0.54		
Mean					
	FSA score	3.03 * (*η^2^* = 0.03)	−2.76 **	HFL	N/A
	Calories	0.76	0.29		N/A
	Sugar	3.27 * (*η^2^* = 0.04)	−2.45 *	Manga, Comic	N/A

Note. *** = *p* < 0.001, ** = *p* < 0.01, * = *p* < 0.05, . = *p* < 0.10. N/A: Due to an inconsistent pattern when adding the “categories” variable, we decided not to report contrasts for the mean outcome variables within food categories.

## Data Availability

Aggregated data is available upon request.

## Appendix A

**Detailed inferential statistics.**

**Total measurements.**

**Total number of products bought.** First, we calculated an ANOVA with a planned contrast of the label vs. no label conditions over the aggregated data (without the category variable), which showed a significant difference in the total number of products bought, t(349) = 3.32, *p* < 0.001, indicating that more products were bought in the label conditions than in the control condition. The Tukey-adjusted post hoc tests showed differences between the comic label and the control condition, t(349) = 3.35, *p* < 0.01, as well as between the Nutri-Score and the control condition, t(349) = 2.98, *p* < 0.05. The post hoc tests between the control and the other labels (HFL and manga) were not significant (both t < 2.13, *p* > 0.21), and neither were the between-label comparisons (t < 1.38, *p* > 0.64).

Second, to assess differences between the food categories, a repeated-measures ANOVA was calculated with the planned contrast of the label vs. no label conditions in each category. The overall label vs. no label contrast was identical when the factor category was added for all dependent measurements when not stated otherwise. The results indicate differences in the categories bread, dairy, veggie, and pasta (all *t* > 3.06, *p* < 0.01) but not for the other categories (all *t* < 1.83, *p* > 0.06), indicating that in the label conditions people bought more bread, dairy, veggie, and pasta products than in the no label condition (see Figure 4).

**Total number of healthy products bought.** The ANOVA with the label vs. no label contrast showed a difference in the number of healthy products bought, *t*(349) = 5.71, *p* < 0.001. Moreover, the Tukey-adjusted post hoc tests between the labels and no label condition were significant (all *t* > 3.80, *p* < 0.01) but not between the labels (all *t* < 1.29, *p* > 0.69), indicating that *more healthy* products were bought *in each of the label conditions* than in the control condition. The contrasts in the repeated-measures ANOVA showed differences in all categories (all *t* > 2.36, *p* < 0.05) but sweets, as in the category sweets the were no healthy products (all sweets were unhealthy).

**Total FSA score bought.** The ANOVA with the planned contrast of the label vs. no label conditions showed a significant difference regarding the total FSA score bought, *t*(349) = 2.30, *p* < 0.05, indicating that the sum of all FSA scores of all the products bought was higher in the label conditions than in the control condition. Tukey-adjusted post hoc tests showed no difference between the conditions (comic vs. control condition; *t*(349) = 2.57, *p* = 0.08, all other *t* < 2.20, *p* > 0.18). The contrasts in the repeated-measures ANOVA showed differences in the categories; bread, dairy, veggie, and pasta (all *t* > 2.99, *p* < 0.01) but not in the other categories (all *t* < 1.45, *p* > 0.14).

**Total calories bought.** The ANOVA with a planned contrast of the label vs. no label conditions showed a difference regarding the total calories bought, *t*(349) = 2.78, *p* < 0.01, indicating that the *total calories* of the products bought was *higher in the label conditions* than in the control condition. Tukey-adjusted post hoc tests showed a difference between the comic label and the control condition, *t*(349) = 2.87, *p* < 0.05, but not for any others (Nutri-Score vs. control; *t*(349) = 2.62, *p* = 0.069, all other *t* < 1.74, *p* > 0.41). The contrasts in the repeated-measures ANOVA showed differences in the categories bread, dairy, veggie, and pasta (all *t* > 2.91, *p* < 0.01) but not in the other categories (all *t* < 1.65, *p* > 0.10).

**Total sugar bought.** The ANOVA with a planned contrast of the label vs. no label conditions showed no significant difference regarding the total sugar bought, *t*(349) = 0.53, *p* = 0.59.

**Mean measurements.**

**Mean FSA score bought.** The ANOVA with a planned contrast of the label vs. no label conditions showed a significant difference in the mean FSA score bought, *t*(349) = −2.76, *p* < 0.01. The Tukey-adjusted post hoc tests showed a difference between the control condition and the HFL, *t*(349) = 3.43, *p* < 0.01, but no difference between the control and the other conditions (all *t* < 2.16, *p* > 0.19). The label vs. no label contrast changed in the repeated-measures ANOVA due to the zeros as missing values, *t*(349) = 0.29, *p* = 0.98, and therefore no contrasts on a category level were calculated.

**Mean calories bought.** The ANOVA with a planned contrast showed no difference in the mean calories bought between the label and control conditions, *t*(349) = 0.29, *p* = 0.78.

**Mean sugar bought.** The ANOVA with a planned contrast showed a difference in the mean sugar bought between the label and control conditions, *t*(349) = −2.45, *p* < 0.05, indicating that the sugar content per product bought was lower in the label conditions than in the control condition. Tukey-adjusted post hoc tests showed a significant difference between the comic and control groups, *t*(349) = 2.82, *p* < 0.05, and the manga and control groups; *t*(349) = 2.81, *p* < 0.05, but not the others (all *t* < 2.25, *p* > 0.16). The label vs. no label contrast changed in the repeated-measures ANOVA due to the zeros as missing values, *t*(349) = 1.10, *p* = 0.27, and therefore no contrasts on a category level were calculated.

## Appendix B

**Table A1:** Detailed product list.

Category	Product	kcal per 100 g	Sugar in g per 100 g	HFL Category	Nutri-Score Category	Sugar Label Category
beverage	apple juice	46	10	orange	C	red
	lemonade	1	0	green	B	green
	coke	42.4	10.6	red	E	red
	still water (Evian)	0	0	green	A	green
	iced tea	28	7	red	D	red
	sparkling water	0	0	green	A	green
	multivitamin juice	59	12	red	D	red
	orange juice	44	9	orange	C	red
	lemonade (Orangina)	38	8.9	red	E	red
	soft drink (Rivella Red)	36	9	red	E	red
	apple spritzer	28	6	red	D	orange
bread	wholegrain cracker (Blévita blau)	463	2.5	orange	C	green
	plaited loaf	305	4	orange	C	orange
	wreath bread	264	3	green	B	orange
	crispbread (Microc)	389	7	orange	C	orange
	pumpernickel	201	6.9	green	B	orange
	rice cake	384	0.5	green	A	green
	dark bread	238	3	green	B	orange
	toast	257	4	green	B	orange
	dark twisted bread	266	2.5	green	A	green
	rusk	424	16	orange	C	red
meat	bratwurst	237	1	red	D	green
	chipolata	233	0.5	red	D	green
	minced meat	189	1	green	B	green
	burger patties	235	1	orange	C	green
	chicken	175	1	orange	C	green
	chicken breast	102	1	green	A	green
	beef strips	130	1	green	A	green
	beef steak	119	1	green	A	green
	pork chops	131	1	green	B	green
dairy	brie	288	0.5	red	D	green
	eggs	142	0.3	green	A	green
	Emmentaler	397	0.1	red	D	green
	grated cheese (Grana Padano)	386	0.5	red	D	green
	Gruyère	405	0.1	red	D	green
	cheese noodles	272	2	orange	C	green
	mozzarella	264	1	orange	C	green
	ricotta	169	4	orange	C	orange
	semi-skimmed milk	57.4	5	green	B	orange
	Tilsiter	385	0.5	red	D	green
	whole milk	66.4	5	green	B	orange
	cream	337	3	red	D	orange
veggie	apples	55	11.6	green	A	orange
	bananas	95	17.2	green	A	red
	mushrooms	22	0.3	green	A	green
	iceberg lettuce	16	1.6	green	A	green
	cucumber	13	1.9	green	A	green
	carrots	38	6.4	green	A	orange
	potatoes	76	0.7	green	A	green
	butterhead lettuce	14	1	green	A	green
	red bell peppers	32	5.1	green	A	orange
	tomatoes	21	3.2	green	A	orange
	grapes	69	15	green	A	red
	lemons	22	2.5	green	A	green
Pasta (and rice)	basmati rice	356	0.5	green	A	green
	carbonara sauce	166	2.5	red	D	green
	gnocchi	321	10	orange	C	orange
	macaroni	361	2.5	green	A	green
	jasmine rice	355	0.5	green	A	green
	penne rigate	361	2.5	green	A	green
	pesto	523	0.8	red	D	green
	risotto	348	0.5	green	A	green
	spaghetti	352	2.5	green	A	green
	spaetzle	165	0.6	green	B	green
	fusilli	361	2.5	green	A	green
	tomato sauce	61.5	7	green	A	orange
	tortellini	209	2	green	B	green
	wild rice	362	0.5	green	A	green
snacks	cracker	519	6	red	D	orange
	peanuts	185	1.6	green	A	green
	almonds	601	3.7	green	A	orange
	potato chips salted	552	0.8	orange	C	green
	olives	231	0	orange	C	green
	red pepper potato chips	544	5	orange	C	orange
	pistachios	611	6.3	orange	C	orange
	potato chips (Pombären)	513	1.2	red	D	green
	salted sticks	373	1	orange	C	green
sweets	chocolate bar (Branches)	541	54	red	E	red
	gummy bears	343	45.6	red	D	red
	raspberry roulade	346	38	red	D	red
	chocolate bar (Kinderriegel)	564	53.3	red	E	red
	madeleines	480	27	red	D	red
	marble cake	408	24	red	D	red
	marshmallows	324	65	red	D	red
	milk chocolate	555	50	red	E	red
	nut chocolate	541	9	red	D	orange
	nut stick cookies	466	34	red	D	red
	butter cookies (Petit Beurre)	471	22	red	D	red
	chocolate cake	465	29	red	D	red
	dark chocolate	541	0.7	red	D	green
	marmalade cookies	489	32	red	E	red
	caramel bites (Toffifee)	516	48.5	red	E	red
	jelly gum (Trolli Bizzl)	334	58	red	D	red
frozen	mini wraps	265	2.5	red	D	green
	lasagna	151	2.5	green	B	green
	mini pies	341	1.5	red	D	green
	pizza baguette	214	3.4	red	D	orange
	pizza	226	3	orange	C	orange
	pizza with ham	222	3	orange	C	orange
	ham puffs	278	2.6	red	D	orange
